# Willingness-to-use and preferences for model-informed antenatal doses: a cross-sectional study among European healthcare practitioners and pregnant women

**DOI:** 10.3389/fphar.2024.1403747

**Published:** 2024-08-15

**Authors:** C. J. M. Koldeweij, A. C. Dibbets, M. Ceulemans, L. C. de Vries, B. D. Franklin, H. C. J. Scheepers, S. N. de Wildt

**Affiliations:** ^1^ Division of Pharmacology and Toxicology, Department of Pharmacy, Radboud University Medical Center, Nijmegen, Netherlands; ^2^ Department of Obstetrics and Gynaecology, Maastricht University Medical Centre, Maastricht, Netherlands; ^3^ Clinical Pharmacology and Pharmacotherapy, Department of Pharmaceutical and Pharmacological Sciences, KULeuven, Belgium; ^4^ IQ Health, Radboud University Medical Center, Nijmegen, Netherlands; ^5^ L-C&Y, KU Leuven Child and Youth Institute, Leuven, Belgium; ^6^ Teratology Information Service, Netherlands Pharmacovigilance Centre Lareb, S’Hertogenbosch, Netherlands; ^7^ Centre for Medication Safety and Service Quality, Imperial College Healthcare NHS Trust, London, United Kingdom; ^8^ Department of Practice and Policy, UCL School of Pharmacy, London, United Kingdom; ^9^ Grow, School for Oncology and Reproduction, Maastricht, Netherlands; ^10^ Department of Paediatric and Neonatal Intensive Care, Erasmus MC-Sophia Children’s Hospital, Rotterdam, Netherlands

**Keywords:** dose, pregnancy, pharmacokinetic models, acceptability, implementation

## Abstract

**Background:** Physiological changes in pregnancy may affect drug safety and efficacy, sometimes requiring dose adjustments. Pregnancy-adjusted doses, however, are missing for most medications. Increasingly, pharmacokinetic models can be used for antenatal dose finding. Given the novelty of this technique and questions regarding dose credibility, the acceptability of model-informed antenatal doses should be explored.

**Objective:** We aimed to assess the willingness-to-use and preferred features for model-informed antenatal doses among healthcare practitioners (HCPs) and pregnant women in European countries.

**Methods:** A cross-sectional, web-based study drawing on two open surveys was performed between 8 September and 30 November 2022. Each survey comprised statements drawn from prior focus groups, associated with Likert-scales. Themes included respondents’ information needs, search behaviours along with their willingness-to-use and preferred features for model-informed antenatal doses. The surveys were disseminated through professional societies, pregnancy websites and social media. A descriptive analysis was performed.

**Results:** In total, 608 HCPs from different specialties and 794 pregnant women across 15 countries participated, with 81% of respondents across both groups in the Netherlands or Belgium. Among pregnant women, 31% were medical professionals and 85% used medication during pregnancy. Eighty-three percent of HCPs found current antenatal pharmacotherapy suboptimal and 97% believed that model-informed antenatal doses would enhance the quality of antenatal care. Most HCPs (93%) and pregnant women (75%) would be willing to follow model-informed antenatal doses. Most HCPs desired access to the evidence (88%), including from pharmacokinetic modelling (62%). Most pregnant women (96%) wanted to understand antenatal dosing rationales and to be involved in dosing decisions (97%).

**Conclusion:** The willingness-to-use model-informed antenatal doses is high among HCPs and pregnant women provided that certain information needs are met.

## Introduction

Physiological changes in pregnant women’s bodies may require dose alterations for certain medications (Westin et al., 2018). Increased plasma volume, augmented renal filtration and altered liver metabolism are examples of pharmacokinetic changes in pregnancy that may potentially result in altered efficacy and safety, requiring dose adjustments for certain medications (Pariente et al., 2016). However, while internationally, over 80% of pregnant women use medication (Lupattelli et al., 2014), the evidence to support antenatal dosing is often lacking (Howard et al., 2018; Stock and Norman, 2019). In the absence of dosing guidance for most medications (Laroche et al., 2020), antenatal dose selection often occurs *ad hoc* in a clinical setting (Sportiello and Capuano, 2023). When prescribing medications to pregnant women, clinicians frequently opt for doses recommended for non-pregnant adults, or reduce doses out of concern for fetal harm (Westin et al., 2018). Given pregnancy-induced changes in pharmacokinetics, for some medications, this may lead to inappropriate maternal and/or fetal exposures potentially affecting therapeutic goals (Westin et al., 2018). For example, reduced exposure to lamotrigine has been observed in pregnancy, in some cases coinciding with increased seizure frequency requiring dose adjustments for seizure control (Goo et al., 2024). The lack of well-researched antenatal doses may thus potentially result in inadequate treatment of maternal and/or fetal disease, as well as potential harm (Jobe et al., 2021), representing a large unmet need for pregnant women and their unborn children (Van Calsteren et al., 2016; Stock and Norman, 2019).

Despite ongoing efforts to increase the enrolment of pregnant women in clinical research, pregnancy-specific data on medication pharmacokinetics, efficacy and safety remain limited given the routine exclusion of pregnant women from drug development research over the past decades in the wake of the thalidomide scandal (Stock and Norman, 2019; Ren et al., 2021; Sportiello and Capuano, 2023). In this context, the emergence of model-informed dosing approaches offers a promising means to supplement the evidence base for antenatal dosing (Abduljalil and Singh, 2020; Chaphekar et al., 2020). Establishing model-informed dosing recommendations may reduce the need to expose additional pregnant women and unborn children to potentially harmful drugs by exploring the impact of pregnancy on maternal and fetal drug exposures.

Pharmacokinetic models include both physiologically-based (PBPK) and population-based pharmacokinetic (pop-PK) models. PBPK models incorporate pregnancy-induced changes in medication absorption, distribution, metabolism and elimination to inform dose selection throughout pregnancy. Pop-PK models, on the other hand, describe the pharmacokinetics of a medication in a given population drawing on concentration samples and the exploration of co-variates to account for individual variability. The accuracy of model predictions can be verified with limited pharmacokinetic data from pregnant women who routinely use medication and from their newborns, where available. For models with sufficient data for validation, and demonstrating adequate predictive performance, the suitability of alternative dose regimens can be investigated (Abduljalil and Singh, 2020; Chaphekar et al., 2020).

The use of pharmacokinetic models alongside clinical and animal data to support antenatal dosing is currently being explored as part of project MADAM (Model-Adjusted Doses for All Mothers), an international initiative seeking to establish proof-of-concept for model-informed antenatal doses (Koldeweij et al., 2024a). Drawing on pharmacological data alongside other considerations for implementation, dose recommendations formulated through this approach will be subject to endorsement by a multidisciplinary committee of experts and patients before dissemination for use by healthcare practitioners (HCPs) and pregnant women.

Given the novelty of the proposed dosing approach, and questions regarding the credibility of model-informed antenatal doses among clinicians and patients along with other potential barriers to implementation (Darwich et al., 2017), successful clinical application requires understanding the acceptability of this methodology among targeted end-users. The aim of this cross-sectional study, therefore, was to assess the willingness-to-use and the preferred features for model-informed antenatal doses according to HCPs and pregnant women.

## Materials and methods

### Definitions

Model-informed antenatal doses, as outlined in this study, referred to medication doses tailored for pregnant women and/or their unborn children, derived from pharmacokinetic (either pop-PK or PBPK) simulations, alongside clinical and/or animal data. The latter doses and underlying evidence should be reviewed by a multidisciplinary committee of experts and patients before endorsement for clinical use (Koldeweij et al., 2024a).

### Questionnaire design

This cross-sectional study was aimed towards the two main groups of stakeholders involved in shared decision-making on antenatal dosing, namely, HCPs and pregnant women. Two questionnaires, one for each targeted group, were designed (Supplementary Material S1). Both questionnaires comprised demographic questions followed by statements drawn from focus groups and interviews on the perceived barriers and facilitators for model-informed dosing in pregnancy previously conducted among HCPs and pregnant women in the Netherlands (Koldeweij et al., 2024). The proposed model-informed dosing approach was introduced briefly on the survey’s opening page, with more details provided later in the survey. Explored themes in both surveys included respondents’ knowledge, information needs and search behaviours with regards to medication dosing in pregnancy, their views on important considerations for dosing, their willingness-to-use model-informed antenatal doses and their information needs and preferred features in this regard. HCPs were additionally asked about their current dosing practices. The questionnaire for HCPs comprised 39 questions and the questionnaire for pregnant women 27 questions. Each statement was associated with either a four-point Likert scale (strongly agree, somewhat agree, somewhat disagree, fully disagree) or a dichotomized response (agree, disagree). Some statements additionally included a ‘not applicable’ response. Optional text boxes were included for respondents to explain their answers. The questionnaires were designed for access on laptops and smartphones and were available in English and Dutch. Questionnaire content and language was tailored in complexity for each group, drawing on experience gained from the focus groups that informed this survey. Castor EDC (https://www.castoredc.com/) was used for questionnaire design and administration.

### User-testing: HCP, patient and public involvement

Each questionnaire was user-tested for aspects including content, length (aiming to not exceed 10 minutes), user-friendliness, intelligibility and technical functioning. User-testing was performed by at least three clinicians (for HCP surveys) and three pregnant or recently pregnant women (for surveys targeting pregnant women) who were fluent in the survey language (Supplementary Material S2). The surveys were additionally tested by three experts in online surveys on related topics. Several iterations of the questionnaires were made to address user feedback.

### Eligibility and recruitment

HCPs were eligible if they were practicing in a European country (OECD). Women were eligible if they were currently pregnant or had been pregnant in the last 3 years, if they were more than 18 years old and resided in a European country. All participants had to be proficient in English or Dutch. The surveys were disseminated through open hyperlinks shared with professional societies of HCPs involved in making decisions or providing information on dosing in pregnancy, including physicians across various specialties, pharmacists, pharmacologists and midwives. Additionally, they were shared on websites and social media for pregnant women across European countries, primarily the Netherlands and Belgium. The links to both surveys were additionally placed on the websites of several national teratology information services (TIS). Posters and flyers with QR codes were left at a small number of outpatient clinics in the Netherlands. No incentive for participation was offered. More information on dissemination channels can be found in Supplementary Material S3. Other than the TIS websites, the links to each survey were disseminated through separate channels so that it was unlikely that a respondent would participate in both surveys. The surveys remained active between 8 September and 30 November 2022.

### Statistical analysis

No sample size was pre-emptively determined. Given the open survey dissemination a response rate could not be calculated, and the lack of prior cross-sectional studies on a similar topic made it difficult to estimate the distribution of values for each survey item *a priori*. All surveys for which demographic information was available and at least one statement had been rated were included. Survey item ratings were analysed individually. The ratings were dichotomized, combining “strongly agree” and “somewhat agree” into the category “agree” and employing a similar method for “disagree.” Statements with a “non-applicable” response were trichotomized. For relevant questions, we broke down the results by five subgroups of HCPs: community pharmacists, hospital pharmacists or clinical pharmacologists, obstetricians-gynecologists, general practitioners and other medical specialists. The analysis was descriptive, using Excel 2013 version 16.68. “Survey participation” describes the ratio of participants who filled in any information divided by the number of survey visitors. “Survey completion” corresponds to the ratio of users finishing the survey divided by those agreeing to participate.

### Ethics and reporting

The study protocol was assessed by the Medical Ethics committee of the Radboud University Medical Centre (2021-13417) and was not subject to the Medical Research Involving Human Subjects Act. Information on the survey and underlying goals were shared with potential respondents in the introductory pages of the survey. Potential respondents were then asked if they wished to participate and agreed with the use of their anonymous data (yes or no button with exit option, Supplementary Material S1). Participation beyond this point implied informed consent. Responses were not identifiable, IP addresses were not recorded.

It is recognized that not all women were pregnant at the time of completing the survey and that not all pregnant people identify as women (Rioux et al., 2022); however for brevity we use the term ‘pregnant women’ to describe our target population. Reporting of the study was guided by the CHERRIES checklist (Supplementary Material S4) (Eysenbach, 2004).

## Results

### Characteristics of respondents

In total, 608 HCPs from 15 countries and 794 women from 12 countries participated. Among HCPs (Table 1), 56% worked in the Netherlands and 25% in Belgium. HCPs comprised 37% pharmacists or clinical pharmacologists, 26% obstetricians-gynecologists, 11% midwives and 26% HCPs from other specialties. HCPs had a median of 13 years of professional practice and worked across a variety of settings, ranging from primary care to academic hospitals. Among pregnant women (Table 2), 49% resided in the Netherlands and 32% in Belgium. A large majority (96%) described themselves as white. Forty-three percent of women had obtained a university degree and 31% were healthcare professionals. 33% were currently pregnant. Eighty-five percent of women had used medication during their pregnancy, and 38% had a chronic or recurring condition that required medication. The survey participation rates were 14% among HCPs and 35% among pregnant women. Survey completion rates were 70% for HCPs and 68% for pregnant women (Supplementary Material S5).

**TABLE 1:** Demographic characteristics of healthcare practitioners.

Demographic information	Respondents (n = 608) (%)
Gender	
Female Male Other Prefer not to answer	466 (76) 133 (22) 2 (<1) 6 (2)
Age	
Age (years)	23–72 (median = 38)
Country	
Albania Austria Belgium Croatia France Germany Greece Hungary Ireland Lithuania Netherlands Norway Sweden Switzerland United Kingdom	2 (<1) 1 (<1) 152 (25) 1 (<0.1) 6 (1) 5 (1) 2 (<1) 1 (<1) 7 (1) 1 (<1) 341 (56) 4 (1) 45 (7) 1 (<1) 39 (6)
Specialty	
Hospital pharmacists or clinical pharmacologists* Community pharmacists General Practitioners Midwives Obstetricians-gynecologists Other medical specialists**	75 (12) 152 (25) 59 (9) 70 (11) 154 (25) 98 (16)
Level of training	
In training Specialist, consultant or fully trained pharmacists or midwife	73 (12) 535 (88)
Years of work experience since obtaining your degree	
Years of work experience since obtaining your degree	0–45 (median = 13)
Work setting	
Academic hospital Community hospital Community pharmacy Primary care Other***	165 (27) 178 (29) 145 (24) 102 (17) 18 (3)

*Clinical pharmacologists counted in this section indicated clinical pharmacology as their sole specialty. One community pharmacist, two general practitioners and six other medical specialists were additionally trained as clinical pharmacologists. **Other medical specialists include anesthesiologists, general medical doctors, cardiologists, internists, pediatricians, neurologists, psychiatrists, geneticists, dermatologists, gastroenterologists, pharmaceutical doctors, and public health physicians. ***Other settings include private practices, universities, pharmaceutical companies and research organisations.

**TABLE 2:** Demographic characteristics of pregnant women.

Demographic information	Respondents (n = 794) (%)
Country	
Belgium France Germany Greece Hungary Ireland Italy Malta Netherlands Spain Sweden United Kingdom	252 (32) 6 (<1) 3 (<1) 3 (<1) 1 (<1) 84 (11) 1 (<1) 1 (<1) 385 (48) 5 (<1) 33 (4) 20 (3)
Age	
Age (years)	21–52 (median = 36)
Ethnicity	
African or Black African + White African or Black + Asian + White Asian Asian + White Hispanic or Latino Hispanic or Latino + African Hispanic or Latino + White White Prefer not to disclose	1 (<1) 1 (<1) 2 (<1) 9 (1) 6 (<1) 3 (<1) 7 (<1) 2 (<1) 761 (96) 2 (<1)
Educational degree or level of schooling	
Primary school or middle school High school Technical/vocational training University degree Doctoral degree Prefer not to disclose	57 (7) 92 (12) 303 (38) 326 (41) 15 (2) 1 (<1)
Trained or works as a medical professional	
Trained or works as a medical professional Medical doctor Midwife Nurse Pharmacist Other	245 (31) 45 (6) 14 (2) 74 (9) 53 (7) 59 (7)
Currently pregnant	
Currently pregnant	261 (33)
Used or uses medication during pregnancy	
Yes Prescription medication Over-the-counter medication No Considered using medication Prefer not to say	675 (85) 554 (70) 309 (39) 116 (15) 30 (4) 3 (<1)
Long term or recurring condition that requires medication	
Yes	302 (38)

### Current dosing practices, information needs and knowledge of HCPs

**FIGURE 1:**
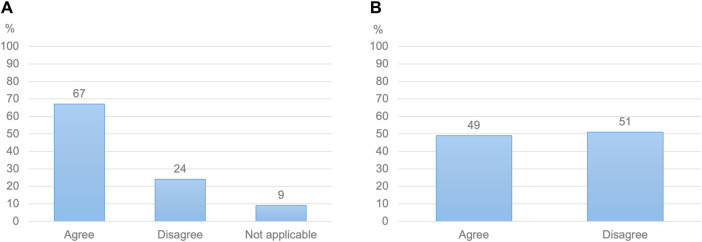
**(A)** Healthcare practitioners’ views on whether fetal safety is a bigger concern than maternal efficacy when prescribing or advising medication to a pregnant woman (N = 592) **(B)** Pregnant women’s views on whether the efficacy of a medication holds as much importance as its safety for their baby (N = 772).

Just over half of HCPs (56%) indicated regularly adjusting doses for pregnant patients (Table 3). This proportion varied by medical specialty, from 48% of obstetrician-gynecologists to 74% among other medical specialists (Supplementary Material S6). Most HCPs (73%) reported discussing medication doses with pregnant patients. While 83% of HCPs indicated concerns about suboptimal pharmacological care for pregnant women, two-thirds prioritized fetal safety over maternal efficacy when prescribing antenatal medications (Figure 1). Virtually all HCPs (97%) indicated that better antenatal dosing information was needed. The same proportion of HCPs believed that an evidence-based resource with model-informed doses would greatly enhance the quality of antenatal pharmacotherapy. On the other hand, HCPs reported limited comprehension of pharmacokinetics in pregnancy. Only 34% of obstetrician-gynecologists, 33% of other medical specialists, 39% of general practitioners and 51% of clinical pharmacists and pharmacologists considered their understanding of pharmacokinetics in pregnancy and the impact on medication safety and efficacy sufficient. Additionally, HCPs reported limited knowledge of pharmacokinetic models. While two-thirds of HCPs agreed that they knew what such models entailed, that only 21% *strongly* agreed with this statement (Supplementary Material S5).

**TABLE 3:** Healthcare practitioners’ information needs and perspectives on model-informed antenatal doses.

Sections	Statements	Number (N)	(Strongly) agree (%)	N.a. (%)
First thought on model-informed doses	I would be willing to follow antenatal doses that are primarily based on evidence from computer models	609	92	1
Current dosing practices and knowledge	Fetal safety is a bigger concern than maternal efficacy when I give advice on medication to a pregnant woman	592	67	9
	I regularly adjust or recommend adjustments in the doses of pregnant patients	581	56	4
	I generally discuss my considerations for choosing a dose with my pregnant patients	573	73	5
	I have a good enough understanding of how pregnancy influences medication PK and of how this may alter safety and efficacy	570	38	1
	I know what pharmacokinetic models entail	571	67	-
Views on available dosing information	There is a need for better information on dosing in pregnancy	557	97	-
	I am concerned that pregnant women receive suboptimal pharmacological care	557	83	-
	The availability of an evidence-based PF would greatly enhance the quality of care for pregnant women	557	97	-
Acceptability of model-informed doses	I am willing to prescribe a higher dose to a pregnant woman:			
	- If this is recommended by the PF, including if the main evidence stems from pharmacokinetic models	532	85	4
	- If this is recommended by the PF, including if fetal exposure to the medication is unknown	526	49	4
	I would be more willing to follow model-informed doses if the methods used is endorsed by recognized institutions	525	96	1
Preferred features of pregnancy-adjusted doses: information needs, access and selection of medicines	I would like the following information to be included as part of model-informed antenatal dose recommendations:			-
	- The balance between maternal and fetal risks and benefits	506	97	-
	- Physiological changes in pregnancy and how they affect medication PK	506	91	-
	- Information on fetal exposure	506	92	-
	- Information on fetal safety	506	98	-
	- Recommendations on how to adjust doses for an individual patient	506	97	-
	- Recommendations for detecting underdosing or toxicity	505	94	-
	I am likely to consult information on the evidence behind a dose in pregnancy	504	88	-
	If yes, what I would like to know:			
	- What the quality of the underlying evidence is*	443	98	-
	- I would like to have access to the evidence itself*	443	78	-
	- If the evidence for a dose comes from a PK model, I would like to access information on the model	443	62	-
	If ‘agree’ to the above: what I would like to know:			
	- The general assumptions of the model*	275	96	-
	- Information on how fetal exposure was determined*	275	93	-
	- Information on model validation*	275	89	-
	- I would like to access the model itself*	275	52	-
	I would like to be able to refer a pregnant woman to online patient information about dosing in pregnancy	499	87	-
	Key considerations for including medications in the PF should be:			
	- The frequency of use of the medication among pregnant women*	499	74	
	- the consequences of underdosing or overdosing in pregnancy*	499	78	

N.a: not applicable; PBPK: pharmacokinetics; *: optional questions. The number and wording of the statements was slightly condensed for inclusion in the table, complete statements can be found in Supplementary Material S1.

### Information needs, search behavior and knowledge of pregnant women

Almost all women (99%) wanted to know which dose to use during pregnancy (Table 4). While sixty-five percent of women had searched information about medication doses while pregnant, only 40% stated that they could easily find clear and helpful information in this regard. Nearly two-thirds of women reported being aware of the potential need for dose adjustments in pregnancy based on their changing bodies. However, only 52% knew that medication doses had generally not been researched in pregnant women. Virtually all women indicated a desire not only to receive information on antenatal dosing (96%) but also to be involved in antenatal dosing decisions by their HCP (96%). Almost half of women (49%) described maternal efficacy and fetal safety as equally important considerations in this regard, with 50% assigning more weight to fetal safety. The remaining 1% assigned more weight to maternal efficacy (Figure 1). Eighty-two percent were open to using a higher dose upon their HCP’s recommendation. Of the remaining 18% who were reluctant to use increased doses, virtually all (98%) cited fetal safety concerns. However, 87% of women would be willing to reconsider if their HCP provided a rationale for altered dosing.

**TABLE 4:** Pregnant women’s information needs and views on model-informed antenatal doses.

Sections	Statements	Number (N)**	(Strongly) agree (%)
Thoughts and information needs on medication use	I would like to know if I can safely use a medication during pregnancy	794	99
	I want to know about the evidence used to determine if I can safely use medication while pregnant	786	93
	I have looked up information on whether I can safely use medication while pregnant	791	94
	If yes: I could easily find clear and helpful information about medication safety during pregnancy	732	53
	The efficacy of the medication I use while pregnant or breastfeeding is as important to me than the safety of this medication for my baby	772	49
	I want to be involved in decisions on which medications I should use during pregnancy together by my HCP	767	96
Thoughts an information needs on medication doses	Before I read the introduction, I was aware that changes in my body during pregnancy can influence the dose of medication I need	744	63
	I am aware that medication doses in pregnancy are often based on studies conducted in nonpregnant people	743	52
	I would like to know which dose I should use during pregnancy	730	99
	I have looked up information about which dose I should use during pregnancy.	725	65
	If yes: I could easily find clear and helpful information about a medication dose during pregnancy	463	40
	I would like to understand why pregnant women may sometimes need a higher or lower dose than women who are not pregnant	716	96
	I would like to have information on how much of the medication I use during my pregnancy goes to my baby	715	96
	I want to be involved in decisions on which dose I should use during pregnancy by my HCP	713	97
	I am willing to use a higher dose during my pregnancy than that I would receive if I was not pregnant if my HCP recommends this	713	82
	If no: If I was advised to use a higher dose, I would be concerned about the safety of this dose for my baby*	127	98
	I would be willing to use an increased dose if my HCP explains the reasons behind this*	127	87
	I am willing to have a blood sample taken before and during my pregnancy if this helps to ensure I receive the right dose	703	97
Your views about the use of computer models	I am willing to use a dose that was determined by computer models alongside the expertise of medical professionals	664	77
	If yes:		
	One reason I am in favour of using models is that they help determine the right dose for treating me effectively during pregnancy*	493	96
	A reason that I am in favour of using models is that they give information on how much of the medication I receive goes to my baby	485	96
Your information needs	I would like to know more about how these computer models work	635	86
	I would like to have more information about how changes in my body during pregnancy affect the amount of medication I need	636	97
	I would like my HCP to share this information with me	628	97
	I would like to get this information through a website	627	95

HCP: healthcare practitioner; *optional questions. **The number of participants who answered the question. The number and wording of the statements was slightly condensed for inclusion in the table, complete statements can be found in Supplementary Material S1.

### Acceptability of a model-informed antenatal doses according to HCPs

**FIGURE 2:**
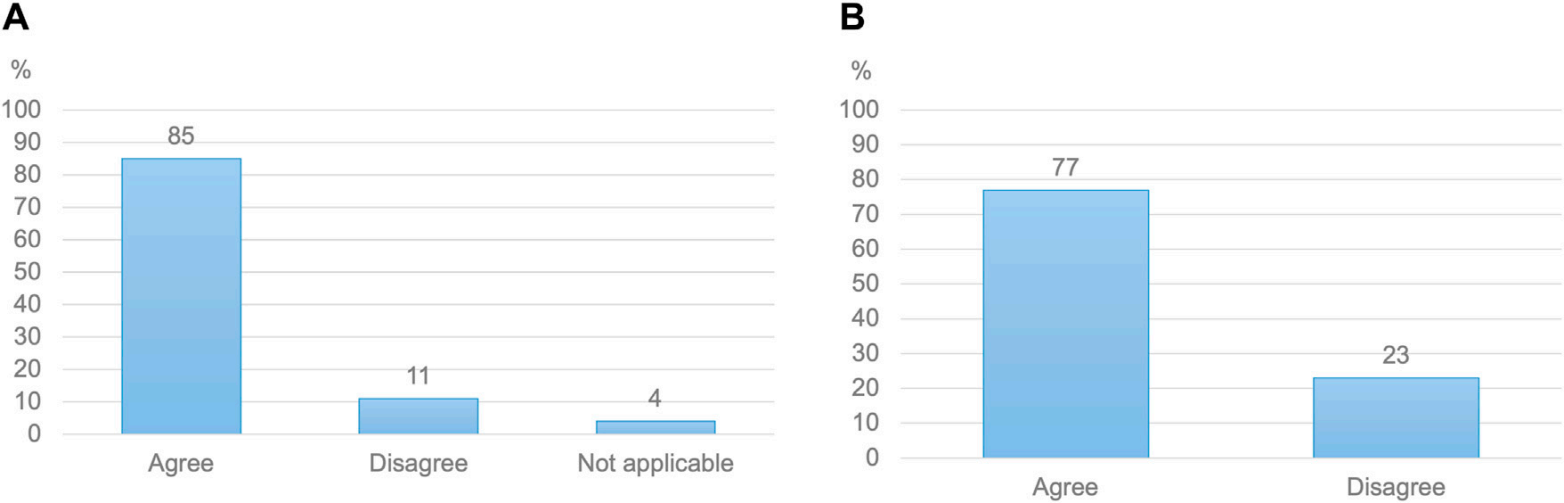
**(A)** Healthcare practitioners’ willingness to follow antenatal doses primarily based on evidence from computer models (N = 532) **(B)** Pregnant women’s willingness to use doses determined by computer models, alongside the expertise of medical professionals (N = 664).

When initially asked, 92% of HCPs stated that they would be willing to follow model-informed antenatal doses. Respondents reiterated this view after receiving more insights on the proposed approach, with 85% of them indicating that they would be willing to advise a higher dose to a pregnant woman based on evidence from pharmacokinetic models (Figure 2). This high willingness-to-use model-informed antenatal doses was found across HCP specialties (Supplementary Material S6). Endorsement of the proposed resource and methods by recognized institutions was perceived as helpful in this view by 96% of HCPs.

### Acceptability of model-informed antenatal doses according to pregnant women

Among women, 77% would be willing to use doses issued based on evidence from computer models (Figure 2). When asked about potential advantages of such models, 96% of pregnant women acknowledged the ability of pharmacokinetic models to guide the selection of more effective maternal doses. A similar proportion welcomed such models’ ability to provide information on fetal exposure. Potential concerns included the lack of individual applicability of model-informed doses, computational errors and insufficient evidence on the predictive ability of models.

### Preferred features for model-informed antenatal doses according to HCPs

HCPs’ key information needs included: information on fetal safety (98%) and on the maternal and fetal risks and benefits of a dose (97%), guidance on considerations for individual dose adjustments (97%) and on how to detect underdosing or toxicity (94%), alongside an overview of the physiological and pharmacokinetic changes that may justify dose alterations (92%). Over half of HCPs (51%) appeared unwilling to follow model-informed antenatal doses without information on fetal exposure. In addition, 88% of HCPs indicated that they would consult evidence on model-informed antenatal doses, starting with information on the quality of the available evidence (97%). If the evidence for a dose stemmed from a pharmacokinetic model, 62% of HCPs would like to access information about the model. Details that HCPs wanted to access in this regard included the general assumptions of the model (94%), information on how fetal exposure was determined (93%), and information on model validation (89%). Just half (52%) of HCPs reported wanting access to the model itself. Lastly, eighty-seven percent of HCPs agreed that information for patients should be provided. Key considerations for integrating medications in the proposed antenatal dosing resource according to HCPs included the frequency of use of the medication in pregnancy (74%) and the consequences of underdosing or overdosing (78%).

### Preferred features of model-informed antenatal doses according to pregnant women

Eighty-six percent of pregnant women desired to know more about how computer models could be used to guide the establishment of pregnancy-adjusted doses. Additionally, 97% of them would like to access information about the relationship between changes in their bodies and their required amount of medication during pregnancy. Pregnant women wanted to access information on model-informed doses and the underlying approach on a website (95%) as well as through their HCP (97%).

## Discussion

### Main findings

This international, cross-sectional study explored the perspectives of HCPs and pregnant women on the acceptability and desirable features of model-informed antenatal doses. Firstly, it revealed that most HCPs and pregnant women deemed the existing information on antenatal dosing inadequate and believed that enhanced information, including from pharmacokinetic models, may greatly improve the quality of maternofetal pharmacotherapy. Secondly, while almost two-thirds of HCPs reported advising or performing antenatal dose adjustments, less than half felt that they had sufficient guidance or knowledge to do so. Thirdly, despite their limited familiarity with pharmacokinetic models, over 90% of HCPs and three-quarters of pregnant women appeared willing to follow model-informed antenatal doses. This contrasted with initial concerns expressed by certain HCPs participants to the focus groups that informed this study regarding the credibility and feasibility of model-informed doses for clinical use. While their information needs differed, both HCPs and pregnant women expressed a desire to better understand dosing rationales, with most participants in both groups wanting to access information on pharmacokinetic models. Other facilitators for the adoption of model-informed antenatal doses according to HCPs included endorsement of the methods by recognized institutions, guidance on applying model-informed antenatal doses for individual patients, and various levels of access to the evidence. A potential barrier related to both groups’ unmet information needs regarding fetal safety, a knowledge gap that may only partly be addressed by pharmacokinetic models according to participants. Lastly, this study highlighted diverging views among HCPs and pregnant women regarding shared decision-making on antenatal dosing. Most pregnant women desired more information and active participation in antenatal dosing decisions. In addition, they tended to value maternal efficacy and fetal safety equally. In contrast, fewer HCPs reported discussing antenatal doses with pregnant women, and most HCPs prioritized fetal safety over maternal efficacy.

### Strengths and limitations

By focusing on the two main groups of stakeholders for a resource with model-informed antenatal doses, this study delivered valuable insights on the acceptability and preferred features for such a resource among potential end-users. Through its broad thematic scope, including HCPs’ dosing practices and HCPs and women’ views on available dosing information, this study was the first to explore the perceived relevance of model-informed antenatal doses against the *status quo*. Although selection bias may be present, as discussed below, the design of the survey questionnaires, drawing on insights from previous focus groups among pregnant women and HCPs in the Netherlands, and the large number of participants likely increased the generalizability of the obtained results.

However, several limitations apply. First, the survey was mainly disseminated in countries where Dutch or English were widely spoken and our findings primarily originated from the Netherlands and the Dutch-speaking part of Belgium. Second, despite the general scope of the dissemination channels used, study participants may have had an above-average degree of knowledge and/or affinity with (model-informed) dosing in pregnancy. For pregnant women especially, limited ethnic diversity, higher-than-average educational levels and an elevated proportion of medical professionals (Centraal Bureau voor de Statistiek, 2023; Hoeveel inwoners hebben een herkomst, 2023; Ministerie van Onderwijs Cultuur en Wetenschap, 2023), may have influenced the insights gathered. However, the incidence of medication use and chronic conditions among pregnant participants aligned well with external data (Lupattelli et al., 2014; Houben et al., 2020; Laroche et al., 2020). Participants’ potentially above-average interest in antenatal dosing and/or pharmacokinetic modelling due to self-selection could either have manifested as a more favorable stance towards model-informed antenatal doses, or conversely, as more skepticism. A cross-sectional study conducted by Nordeng et al. among 1,793 women in Norway found that higher education levels were associated with a significantly increased risk perception regarding antenatal drug use and choosing not to use a drug during pregnancy (Nordeng et al., 2010). The latter pattern was observed in the focus groups that informed this study: individuals with a deeper grasp of pharmacokinetic models, such as clinical pharmacologists, tended to raise more questions about the modelling approach. The lack of questions, in this survey, on challenges associated with data collection for model development and validation (Kluwe et al., 2021), may also have resulted in limited awareness, among participants less familiar with pharmacokinetic modelling, of this concern for model credibility. Questionnaire content may thus have influenced participants’ readiness-to-use model-informed doses, especially among those less knowledgeable on this subject. Other limitations included the exclusion of partners of pregnant women and the absence of formal mechanism to prevent double entries in the survey.

### Interpretation and implications for practice

The limited reports on the clinical implementation of model-informed dosing were expert reviews that examined the use of this approach in non-pregnant populations (Darwich et al., 2017; Hartman et al., 2020; Kluwe et al., 2021). These studies generally focused on technical requirements rather than end-users’ preferences, as explored in this study. Beyond examining the acceptability and preferred features of model-informed antenatal doses, this study clarified stakeholders’ perspectives on antenatal dosing, a poorly studied area. While previous work noted HCPs’ limited awareness of specific antenatal dosing needs (Westin et al., 2018), most participants in our study believed that there was a lack of high-quality information on antenatal doses and felt that their knowledge was insufficient to make informed antenatal dosing decisions. To our knowledge, pregnant women’s information needs and preferences with regards to antenatal dosing have not previously been explored.

Prior research into stakeholder views regarding antenatal pharmacotherapy focused on medication use rather than dosing. A cross-sectional study in Belgium found that many pregnant women sought pharmacological information online but that fewer discussed their findings with a HCP (Ceulemans et al., 2019). An international review reported that most pregnant women searched health information, including on medication, online, although the aspects of medication researched were not described (Sayakhot and Carolan-Olah, 2016). An earlier investigation into HCPs and pregnant women’s perceptions of risks from antenatal medication use found that both groups were highly sensitive to potential risks in the face of scarce evidence (Widnes and Schjøtt, 2017). The importance each group placed on those risks compared to potential benefits from antenatal medication use was not quantified. However, both HCPs and pregnant women in this study were deemed to place undue emphasis on teratogenicity compared to the potential maternal and fetal benefits from medication use according to the study authors, a finding aligning with previous research (Nordeng et al., 2010; Lynch et al., 2018). By contrast, other researchers reported that pregnant women, despite concerns about fetal safety, also cared about the maternal effects of medication (Lynch et al., 2018). This viewpoint was also conveyed by several pregnant participants to the focus groups informing this study. The present study confirmed and quantified the differing views of HCPs and pregnant women regarding the importance of fetal safety compared to potential maternal benefits when making antenatal dosing decisions, with two-thirds of HCPs prioritising fetal safety while nearly half of pregnant women valued both equally. Despite previous anecdotal accounts of a tendency among HCPs to prioritise fetal safety (Widnes and Schjøtt, 2017), this quantified assessment among both groups represents a new finding within the limited research on HCPs’ and pregnant women’s decision-making on antenatal medication, warranting further exploration. The need to better align HCPs’ dosing practices with pregnant women’s preferences was further underlined by both groups’ diverging views regarding shared decision-making on antenatal dosing, another topic that has received little scrutiny.

Model-informed antenatal doses established through the MADAM project will be published and made accessible on the websites of the Dutch Teratology Information Service and the Dutch National Formulary so they can inform clinical practice in the Netherlands (Koldeweij et al., 2024b). Next steps as part of this proof-of-concept will focus on disseminating established doses internationally, for example, through international TISes. Tailored information for HCPs and patients on the approach used and underlying premises will be made available on a separate website (https://melinda-dosing.com/).

Although model-informed dosing may help deliver better-evidenced doses in pregnancy, this study also suggests that it may not fully address the perceived challenges surrounding antenatal medication. Improving awareness of potentially altered antenatal dosing needs among HCPs and pregnant women, while expanding the evidence on the pharmacokinetics, efficacy and safety of antenatal medications, are also essential. Addressing these gaps may require multiple interventions including more systematic pharmacokinetic studies in pregnancy (Quinney et al., 2023), broader enrolment of pregnant women in clinical trials (Eke et al., 2019; Eke et al., 2021; Ren et al., 2021), education of HCPs and women on pharmacokinetics in pregnancy (Westin et al., 2018), alongside the deployment of evidence-based information resources and shared decision-making aids on antenatal medication use and dosing (Eke et al., 2019). To enhance these efforts, future research may involve exploring the characteristics of healthcare practitioners and pregnant women more or less inclined to follow model-informed doses in pregnancy, and for pregnant women to be involved in antenatal dosing decisions. Insights from this study will be incorporated in the design of an international, model-informed antenatal dosing resource. Lastly, this study highlighted a need to alter risk perspectives associated with antenatal drug use among HCPs and pregnant women. This may entail further exploring how these views are influenced by individual features potentially related to understanding of these risks, particularly among HCPs, and how these views may differ from those of pregnant women. These insights could inform tailored knowledge dissemination efforts including dedicated trainings on risk evaluation and communication for HCPs, both as part of medical school curriculums and continuous education. Insights from this study may also guide the development of enhanced tools for risk communication and shared decision-making integrating pregnant women’s values (Eke et al., 2019; Kennedy et al., 2020)). Such efforts may also help more broadly address a potential disconnect between the expectations and behaviors of HCPs and pregnant women regarding antenatal pharmacotherapy, as revealed by this study.

## Conclusion

Healthcare practitioners and pregnant women demonstrated a high willingness-to-follow model-informed antenatal doses, despite their novelty. A key driver in this regard, was a perceived lack of information on antenatal dosing, believed to contribute to the currently suboptimal quality of care in pregnancy. Both groups desired access to the underlying evidence and dosing rationales. While a model-informed antenatal dosing resource may not resolve all knowledge gaps regarding adequate antenatal pharmacotherapy, participants believed that it could help ensure that pregnant women and their unborn children receive safer and more effective treatments. More broadly, this study highlights the need to further explore and address potential discrepancies between the information needs and preferences of pregnant women, and existing clinical practices regarding antenatal drug decisions.

## Data Availability

The raw data supporting the conclusion of this article will be made available upon request by the authors, without undue reservation.

## Supplementary material

The Supplementary Materials for this article can be found online at: https://www.frontiersin.org/articles/10.3389/fphar.2024.1403747/full#supplementary-material
