# Supplementary material for: Willingness-to-use and preferences for model-informed antenatal doses: a cross-sectional study among European healthcare practitioners and pregnant women
Source: Front Pharmacol. 2024 Aug 15;15:1403747. doi: 10.3389/fphar.2024.1403747 (PMC11358599; doi:10.3389/fphar.2024.1403747)
Supplement: Supplementary file 1 [file DataSheet1.docx]

**Supplemental materials**

1 – Survey questionnaires…….........……………..………………………………………………………………………………….........………………..2

a. Healthcare practitioners……………………………………………………………………………………………………………………...………………2

b. Pregnant women……………………………………………………………………………………………………………………………………………….…5

2 – User testing ……………………………………………………………………………………………………………………..……………….……………….7

3 – Dissemination channels…………………………………………………………………………………………………………..……………….…………8

4 – Checklist for Reporting Results of Internet E-Surveys (CHERRIES)…………….……………..……………..……………….……..…10

5 – Completion rate and participation rate…………………………………………………………………………..……………...…….………….15

6 – Subanalysis per subspecialism…………………………………………………………………..……………….……………………………………..16

7 – Statement ratings…………………………………………………………………………………………………..……………….……………..…………17

a. Healthcare practitioners……………………………………………………………………………………………………………………………….….…17

b. Pregnant women………………………………………..…………………………………………………………………..………………….………………20

**1 – Survey questionnaires**

- 1. **Healthcare practitioners**

*2 answers: disagree, agree.*

*3 answers: disagree, agree, not applicable as part of my work.*

*4 answers: strongly disagree, somewhat disagree, somewhat agree, strongly agree.*

*5 answers: strongly disagree, somewhat disagree, somewhat agree, strongly agree, not applicable as part of my work.*

**Why this survey**

Eligibility and consent

1. Are you a practicing healthcare practitioner (doctor, pharmacist, or midwife): 2 answers (yes and no)
2. After reading the previous information, do you consent for us to use your anonymous responses to the survey as information for setting up the pregnancy formulary and for publications as part of project MADAM: 2 answers (yes and no)

**Demographic questions**

1. What is your gender: 4 answers (female, male, other, prefer not to answer)
2. What is your year of birth: 52 answers (1950-2002)
3. In which country do you work as a healthcare practitioner: (all countries)
4. What is your specialty: 13 answers (anaesthesiologist, clinical pharmacologist, gastroenterologist, general practitioner, gynaecologist and/or obstetrician, internist, midwife, neurologist, paediatrician/neonatologist, pharmacist (hospital), pharmacist (community), psychiatrist, other?
5. Other namely: free text box
6. What is your level of training: 3 answers (specialist, consultant or fully trained midwife, in training to become a specialist, consultant or midwife)
7. How many years of work experience do you have since obtaining your degree: free text box.
8. What is your work setting: academic hospital, community hospital, community pharmacy, primary care, other.
9. Other: free text box.

**Your first thought**

1. I would be willing to follow dose recommendations for pregnant women that are primarily based on evidence from computer models: 5 answers

**The status quo**

1. Fetal safety is a bigger concern than maternal effectiveness when I prescribe medication or give advice on a medication to a pregnant woman that requires medication: 5 answers.
2. I regularly adjust or recommend adjustments in the medication doses of pregnant patients or patients that wish to be pregnant: 5 answers.
3. I generally discuss my considerations for choosing a medication dose with my pregnant patients: 5 answers: 5 answers.
4. I have a good enough understanding of how pregnancy can influence the pharmacokinetics (ADME) of medications and how this may result in an altered safety and effectiveness profile during pregnancy: 5 answers.
5. I know what pharmacokinetic models entail: 4 answers.
6. (Optional) additional comments: free text box.

**Relevance of a pregnancy formulary**

1. There is a need for better information on the fetal safety of medications: 4 answers.
2. There is a need for better information on medication dosing in pregnancy: 4 answers.
3. I am concerned that pregnant women receive suboptimal pharmacological care: 4 answers.
4. The availability of an evidence-based pregnancy formulary would greatly enhance the quality of care for pregnant women: 4 answers.
5. (Optional) additional comments: free text box.

**Acceptability**

1. I am willing to advise or prescribe a higher medication dose to a pregnant woman if this is recommended by the pregnancy formulary, including if the main evidence stems from pharmacokinetic models: 3 answers.
   - 1. (If ‘disagree’ to 1) important concerns for me are: free text box.
2. I am willing to advise or prescribe a higher medication dose to a pregnant woman if this is recommended by the pregnancy formulary, including if the fetal exposure to the medication is unknown: 5 answers.
3. I would be more willing to follow dose recommendations in the pregnancy formulary if the formulary is endorsed by recognized institutions and professional associations: 5 answers.
4. (Optional) additional comments: free text box.

**Dose recommendations**

7 questions

1. Do you agree with the following: I have a preference for framing A over framing B: 4 answers (strong preference for A, slight preference for A, slight preference for B, strong preference for B).

*I would like the following information to be included in the pregnancy formulary:*

1. How the risks associated with the dose recommendation compare with the benefits of said dose recommendation for pregnant women and their unborn child: 4 answers.
2. Relevant physiological changes in pregnancy and how they affect the pharmacokinetics of that medication: 4 answers.
3. Information on the fetal exposure to the medication: 4 answers.
4. Information on the fetal safety of the medication: 4 answers.
5. Recommendations to adjust the recommended dose based on individual characteristics of a pregnant patient: 4 answers.
6. Recommendations for detecting the occurrence of underdosing or toxicity in a pregnant patient receiving the recommended dose: 4 answers.
7. (Optional) other information, namely: free text box.

1 question (7 sub-questions)

1. I am likely to consult information on the evidence behind a dose recommendation in pregnancy: 2 answers.

*What I would like to know (if ‘agree’ to question 1)*

2. What the quality of the underlying evidence is: 4 answers.

3. I would like to have access to the evidence itself: 4 answers.

4. If the evidence for a dose recommendation comes from a pharmacokinetic model, I would like to have access to the information on the model: 2 answers.

*What I would like to know (if ‘agree ‘ to on question 4)*

1. The general assumptions of the model: 4 answers.
2. Information on how fetal exposure was determined: 4 answers.
3. Information model validation: 4 answers.
4. I would like to have access to the model itself: 4 answers.
5. (Optional) other information, namely: free text box.

**Access to the pregnancy formulary**

1. My preference would be to access the pregnancy formulary on a new stand-alone website: 4 answers.
2. My preference would be for the pregnancy formulary to be housed on a website that I already use: 2 answers.
3. (Optional) my preferred website would be: free text box.
4. I would like to have access to the pregnancy formulary via a mobile app: 4 answers.
5. I would like to be able to refer a pregnant woman to online patient information about dosing in pregnancy: 4 answers.
6. (Optional) additional comments: free text.

**Selection of medicines and awareness-raising (optional)**

1. Key considerations for including medications in the pregnancy formulary should be (maximum 2 responses): the frequency of use, the number of specialties prescribing, consequences of underdosing or overdosing, another consideration.
2. Another important consideration for including medications in the pregnancy formulary is: free text box.
3. I would like the following medications or class of medications to be included in the pregnancy formulary: free text box.
4. I recommend the following actions to raise awareness on the pregnancy formulary among relevant stakeholders: free text box.
5. I would like to be part of the editorial board of the envisioned pregnancy formulary: 2 answers (yes or no).
   1. **Pregnant women**

*2 answers: disagree, agree.*

*4 answers: strongly disagree, somewhat disagree, somewhat agree, strongly agree.*

**Why this survey**

Eligibility

1. Are you above 18 years old: 2 answers (yes or no)
   - 1. (If agree to 1) are you currently pregnant: 2 answers (yes or no)
     2. (If agree to 1) have you been pregnant during the last 3 years: 2 answers (yes or no)

Informed consent

1. I agree to the previous uses of my answers and would like to complete the survey: 3 answers (yes, no, prefer not to say)

**What do you think of the information on medication use?**

1. I would like to know whether I can safely use a medication during pregnancy: 2 answers.
2. I would like to know whether I can safely use a medication during breastfeeding: 2 answers.
3. I want to know about the evidence that was used to determine if a medication can safely be used during my pregnancy or breastfeeding: 4 answers.
4. I have looked up information on whether I can safely use a medication during pregnancy or breastfeeding: 4 answers.
5. I could easily find clear and helpful information about the safety of medication during pregnancy or breastfeeding: 4 answers.
6. The effectiveness of the medication I use while I am pregnant or breastfeeding is as important to me than the safety of this medication for my baby: 3 answers (disagree the safety of my baby is more important, disagree the effectiveness of my medication is more important, agree they are equally important)
7. I want to be involved in decisions on which medication I should use during pregnancy or breastfeeding together by my healthcare practitioner: 4 answers.

**What do you think of the information on medication doses?**

Before this survey – 2 questions

1. Before I read the introduction of this survey, I was aware that changes in my body during pregnancy can influence the dose of medication I need: 4 answers.
2. I am aware that the doses of medication used in pregnant women are often based on research from studies conducted in people who are not pregnant: 4 answers.

Your information needs – 6 questions

1. I would like to know which medication dose I should use during pregnancy: 4 answers.
2. I would like to know which medication dose I should use during breastfeeding: 4 answers.
3. I have looked up information about which dose of medication I should use during pregnancy: 2 answers.
   - 1. (If agree to 3) I could easily find clear and helpful information about a medication dose during pregnancy: 4 answers.
4. I would like to understand why pregnant women may sometimes need a higher or lower dose of medication than women that are not pregnant: 4 answers.
5. I would like to have information on how much of the medication I use during my pregnancy goes to my baby: 4 answers.
6. I want to be involved in decision on which dose of a medication I should use during pregnancy by my healthcare practitioner: 4 answers.

Adjusted doses – 2 questions

1. I am willing to use a higher dose of medication during my pregnancy than that I would receive if I was not pregnant if my healthcare practitioner recommends this: 2 answers.

(If no to question 1) if I was advised to use a higher dose, I would be concerned about the safety of this dose for my baby: 4 answers.

1. (Optional) I have other concerns, namely: free text box.
2. I would be willing to use an increased dose of medication if my healthcare practitioner explains the reasons behind the chosen dose: 4 answers.
3. I am willing to take a small blood sample taken before and during my pregnancy if this helps to make sure that I receive the right medication dose for treating my condition: 4 answers.

**How do you feel about the use of computer models?**

1. I am willing to use a medication dose that was determined by computer models alongside the expertise of medical professionals: 2 answers.
2. (Optional) my concerns are: free text box.

(If not to question 1) one reason that I am in favour of using computer models is that they help determine the right medication dose for treating me effectively during pregnancy: 4 answers.

1. Another reason that I am in favour of using such models is that they give information on how much of the medication I receive goes to my unborn child: 4 answers.
2. I would like to know more about how these computer models work: 4 answers.
3. I would like to have more information about how changes in my body during pregnancy affect the amount of medication I need: 4 answers.

**Where do you want to find information?**

1. I would like my healthcare practitioner to share this information to share this information with me: 4 answers.
2. I would like to get this information through a website: 4 answers.

**2 – User testing**

| **Tester** | **Background** | **Tested survey** |
| --- | --- | --- |
| 1 | HCP | HCPs (NL), pregnant women (NL) |
| 2 | HCP | HCPs (NL) |
| 3 | HCP | HCPs (EN), pregnant women (EN) |
| 4 | HCP, researcher (implementation) | HCPs (EN), pregnant women (EN) |
| 5 | Researcher (implementation) | HCPs (NL), pregnant women (NL) |
| 6 | Researcher (online surveys) | HCPs (NL), pregnant women (NL) |
| 7 | HCP, implementation expert | HCPs (NL), pregnant women (NL) |
| 8 | Researcher (modeler) | Pregnant women (NL) |
| 9 | Pregnant woman | Pregnant women (EN) |
| 10 | Pregnant woman | Pregnant women (NL) |
| 11 | Pregnant woman | Pregnant women (NL) |
| 12 | Pregnant woman | Pregnant women (NL) |
| 13 | Pregnant woman | Pregnant women (NL) |

**3 – Dissemination channels**

a. Netherlands

*HCP = Healthcare practitioner, IBD = Inflammatory bowel disease, KNMP = Royal Dutch Pharmacy Society, NVA = Dutch Society for Anaesthesiology, NVKFB = Dutch Society for Clinical Pharmacology and Biopharmacy, NVMDL = Dutch Society for Gastroenterologists, NVN = Dutch Society for Neurology, NVOG = Netherlands Society for Obstetrics and Gynaecology*

| **Association** | **Audience** | **Dissemination method** |
| --- | --- | --- |
| NVOG | Obstetricians-gynaecologists | Mass-email |
| NVA | Anaesthesiologists | LinkedIn post |
| NVN | Neurologists | Post on website |
| NVMDL | Gastroenterologists | Message + link in newsletter |
| KNMP | Pharmacists | Message in newsletter |
| NVKFB | Clinical pharmacologists | Mass mail |
| 24Baby | Pregnant women | Advert |
| Various Facebook groups | Pregnant women | Post + link to survey |
| PRIDE study | Pregnant women | Facebook post |
| Various individuals with an audience of pregnant women or HCPs | Pregnant women and HCP | Poster or link + post on Twitter |
| Crohn and Colitis association Netherlands | HCPs and pregnant IBD patients | LinkedIn post + message in newsletter |
| Asthma association Netherlands | HCPs and pregnant asthma patients | Post on website + newsletter |
| Child health clinics across the Netherlands | HCPs and pregnant women | Posters |

b. International

*EAHP = European Association of Hospital Pharmacists, EnPRNMA = European Network of Paediatric Research at the European Medicines Agency, ESDPPP = European Society for Developmental Perinatal and Paediatric Pharmacology, HCP = Healthcare practitioner, UEG = United European Gastroenterology, UKTIS = United Kingdom Teratology Information Services*

| **Country** | **Organizations** | **Audience** | **Dissemination method** |
| --- | --- | --- | --- |
| Belgium | Professional organizations of family medicine, pharmacy and gynaecology.  Websites: [www.gezondzwangerworden.be](http://www.gezondzwangerworden.be), www.fara.be  Maternity care expertise centres, midwifery practices  Breastfeeding support groups | HCPs and pregnant women | LinkedIn, Twitter, newsletter, local network contacts |
| Ireland | Rotunda Hospital Dublin, Royal College of Surgeons in Ireland, Specialist Perinatal Mental Health Services Ireland | HCPs and pregnant women | Twitter |
| Germany | Teratology information services and social ventures | HCPs and pregnant women | Social media |
| Sweden | Lund University (pharmacovigilance), Start page of Swedish knowledge database regarding drugs and fetal effects: <https://janusmed.se/fosterpaverkan> mailing list midwives and HCPs  Healthportal (pregnant women) | HCPs and pregnant women | Post + link to survey |
| United Kingdom | UKTIS  Royal College of Midwives  Mumsnet | HCPs and pregnant women | Social media of UKTIS, link medsinpregnancy.org |
| Europe | EAHP  ENTIS  ESDPPP  UEG  EnPRNMA  Conect4Children  IMI ConcepTION  European Perinatal Institute of Mental Health | HCPs and pregnant women | Mailing list, social media |

**4 – Checklist for Reporting Results of Internet E-Surveys (CHERRIES)**

| ***Checklist Item*** | ***Explanation*** | ***Section*** |
| --- | --- | --- |
| **Describe survey design** | Describe target population, sample frame. Is the sample a convenience sample? (In “open” surveys this is most likely.) | **Methods – Survey dissemination** |
| **IRB approval** | Mention whether the study has been approved by an IRB. | **Methods – Ethics** |
| **Informed consent** | Describe the informed consent process. Where were the participants told the length of time of the survey, which data were stored and where and for how long, who the investigator was, and the purpose of the study? | - Investigator, purpose, length of the study: in the introduction page of the survey (Appendix 1)  - Data storage: not described |
| **Data protection** | If any personal information was collected or stored, describe what mechanisms were used to protect unauthorized access. | No personal information collected |
| **Development and testing** | State how the survey was developed, including whether the usability and technical functionality of the electronic questionnaire had been tested before fielding the questionnaire. | **Methods – Survey questionnaire, User testing** |
| **Open survey versus closed survey** | An “open survey” is a survey open for each visitor of a site, while a closed survey is only open to a sample which the investigator knows (password-protected survey). | Open survey |
| **Contact mode** | Indicate whether or not the initial contact with the potential participants was made on the Internet. (Investigators may also send out questionnaires by mail and allow for Web-based data entry.) | Mostly online contact, some poster with QR codes – see **Methods, Survey dissemination** |
| **Advertising the survey** | How/where was the survey announced or advertised? Some examples are offline media (newspapers), or online (mailing lists – If yes, which ones?) or banner ads (Where were these banner ads posted and what did they look like?). It is important to know the wording of the announcement as it will heavily influence who chooses to participate. Ideally the survey announcement should be published as an appendix. | **Methods – Survey questionnaire**  **Appendix 5** |
| **Web/E-mail** | State the type of e-survey (eg, one posted on a Web site, or one sent out through e-mail). If it is an e-mail survey, were the responses entered manually into a database, or was there an automatic method for capturing responses? | **Methods – Questionnaire design and Survey dissemination**  Open links placed on websites and newsletters (among other media)  Survey administered via the electronic platform Castor EDC – responses captured automatically |
| **Context** | Describe the Web site (for mailing list/newsgroup) in which the survey was posted. What is the Web site about, who is visiting it, what are visitors normally looking for? Discuss to what degree the content of the Web site could pre-select the sample or influence the results. For example, a survey about vaccination on a anti-immunization Web site will have different results from a Web survey conducted on a government Web site | HCPs: the survey was primarily disseminated through professional societies from the various healthcare specialties involved in prescribing and/or providing information on medication to pregnant women. This allowed to reach a broad variety of HCPs regardless of their level of familiarity and/or expertise in pharmacology during pregnancy. That said, the sample was biased towards the Netherlands and Belgium where a greater number of societies accepted to share the survey.  Pregnant women: General pregnancy websites and social media which did not specifically focus on medication use or specific medical conditions were mostly used for dissemination of the survey towards pregnant women. An exception was the patient-facing pages of national teratology information services which are generally consulted by pregnant women seeking information on medication use and by pregnant women with an above average level of education (source: Dutch Teratology Information Service). This may have induced bias in the sample (more prevalent medication use and/or higher incidence of chronic or recurring medications and higher educational level). No vaccination or anti-immunization pages were used. Again survey dissemination among pregnant women primarily occurred among websites and social media channels in the Netherlands and in Belgium. |
| **Mandatory/voluntary** | Was it a mandatory survey to be filled in by every visitor who wanted to enter the Web site, or was it a voluntary survey? | Voluntary survey |
| **Incentives** | Were any incentives offered (eg, monetary, prizes, or non-monetary incentives such as an offer to provide the survey results)? | None |
| **Time/Date** | In what timeframe were the data collected? | See Methods – Survey dissemination |
| **Randomization of items / questionnaires** | To prevent biases items can be randomized or alternated. | Items were not randomized or alternated. |
| **Adaptive questioning** | Use adaptive questioning (certain items, or only conditionally displayed based on responses to other items) to reduce number and complexity of the questions. | Adaptive questioning was used. See **Methods- Survey Questionnaire** and **Appendix 1** |
| **Number of Items** | What was the number of questionnaire items per page? The number of items is an important factor for the completion rate. | The number of questionnaire items per page varied between 1 and 8 for HCPs (including dependency questions) and between 2 and 6 for pregnant women. |
| **Number of screens (pages)** | Over how many pages was the questionnaire distributed? The number of items is an important factor for the completion rate. | HCPs: 15; pregnant women: 10.  The survey for HCPs was designed and user-tested so as to be completed within 15 minutes; the survey for pregnant women was designed and user-tested so as to be completed within 10 minutes. |
| **Completeness check** | It is technically possible to do consistency or completeness checks before the questionnaire is submitted. Was this done, and if “yes”, how (usually JAVAScript)? An alternative is to check for completeness after the questionnaire has been submitted (and highlight mandatory items). If this has been done, it should be reported. All items should provide a non-response option such as “not applicable” or “rather not say”, and selection of one response option should be enforced. | The questionnaire for HCPs contained 39 questions, out of which 9 were dependency questions and 12 were optional; the questionnaire for pregnant women contained 27 questions, out of which 3 were dependency questions and 2 were optional. Mandatory questions contained the option ‘would rather not say’ or ‘not applicable’ when relevant (i.e. demographic questions or questions about current practices). Respondents could follow their progression in the navigation bar left of the survey (% of survey completed). |
| **Review step** | State whether respondents were able to review and change their answers (eg, through a Back button or a Review step which displays a summary of the responses and asks the respondents if they are correct). | Yes respondents were able to review and change their answers through the use of a Back button. There was no Review step in the questionnaire. |
| **Unique site visitor** | If you provide view rates or participation rates, you need to define how you determined a unique visitor. There are different techniques available, based on IP addresses or cookies or both. | No IP addresses or cookies were used. Given the targeted audiences, the subject of the surveys and the dissemination channels used, it was deemed unlikely that respondents would access the survey twice. Apart from the Teratology Information Service websites, the links to both surveys were disseminated separately so that it was unlikely that a respondent would participate in both surveys |
| **View rate (Ratio of unique survey visitors/unique site visitors)** | Requires counting unique visitors to the first page of the survey, divided by the number of unique site visitors (not page views!). It is not unusual to have view rates of less than 0.1 % if the survey is voluntary. | This data could not be computed by the survey administration platform. |
| **Participation rate (Ratio of unique visitors who agreed to participate/unique first survey page visitors)** | Count the unique number of people who filled in the first survey page (or agreed to participate, for example by checking a checkbox), divided by visitors who visit the first page of the survey (or the informed consents page, if present). This can also be called “recruitment” rate. | This data could not be computed by the survey administration platform. |
| **Completion rate (Ratio of users who finished the survey/users who agreed to participate)** | The number of people submitting the last questionnaire page, divided by the number of people who agreed to participate (or submitted the first survey page). This is only relevant if there is a separate “informed consent” page or if the survey goes over several pages. This is a measure for attrition. Note that “completion” can involve leaving questionnaire items blank. This is not a measure for how completely questionnaires were filled in. (If you need a measure for this, use the word “completeness rate”.) |  |
| **Cookies used** | Indicate whether cookies were used to assign a unique user identifier to each client computer. If so, mention the page on which the cookie was set and read, and how long the cookie was valid. Were duplicate entries avoided by preventing users access to the survey twice; or were duplicate database entries having the same user ID eliminated before analysis? In the latter case, which entries were kept for analysis? | Cookies were not used. |
| **IP check** | Indicate whether the IP address of the client computer was used to identify potential duplicate entries from the same user. If so, mention the period of time for which no two entries from the same IP address were allowed (eg, 24 hours). Were duplicate entries avoided by preventing users with the same IP address access to the survey twice; or were duplicate database entries having the same IP address within a given period of time eliminated before analysis? If the latter, which entries were kept for analysis (eg, the first entry or the most recent)? | Ip addresses were not collected (partly in order to maintain full anonymity of the surveys). |
| **Log file analysis** | Indicate whether other techniques to analyze the log file for identification of multiple entries were used. If so, please describe. | No other method. |
| **Registration** | In “closed” (non-open) surveys, users need to login first and it is easier to prevent duplicate entries from the same user. Describe how this was done. For example, was the survey never displayed a second time once the user had filled it in, or was the username stored together with the survey results and later eliminated? If the latter, which entries were kept for analysis (eg, the first entry or the most recent)? | Open surveys |
| **Handling of incomplete questionnaires** | Were only completed questionnaires analyzed? Were questionnaires which terminated early (where, for example, users did not go through all questionnaire pages) also analyzed? | All questionnaires for which at least one non-demographic question was filled in were included in the analysis. Each question was analyzed separately. |
| **Questionnaires submitted with an atypical timestamp** | Some investigators may measure the time people needed to fill in a questionnaire and exclude questionnaires that were submitted too soon. Specify the timeframe that was used as a cut-off point, and describe how this point was determined. | This functionality was not used. |
| **Statistical correction** | Indicate whether any methods such as weighting of items or propensity scores have been used to adjust for the non-representative sample; if so, please describe the methods. | None were used. |

This checklist has been modified from Eysenbach G. Improving the quality of Web surveys: the Checklist for Reporting Results of Internet E-Surveys (CHERRIES). J Med Internet Res. 2004 Sep 29;6(3):e34 [erratum in J Med Internet Res. 2012; 14(1): e8.]. Article available at [https://www.jmir.org/2004/3/e34](https://www.jmir.org/2004/3/e34/)/; erratum available <https://www.jmir.org/2012/1/e8/>. Copyright ©Gunther Eysenbach. Originally published in the [Journal of Medical Internet](http://www.jmir.org) Research, 29.9.2004 and 04.01.2012.

This is an open-access article distributed under the terms of the Creative Commons Attribution License (<https://creativecommons.org/licenses/by/2.0/>), which permits unrestricted use, distribution, and reproduction in any medium, provided the original work, first published in the Journal of Medical Internet Research, is properly cited.

**5 – Completion rate and participation rate**

Participation rate

*Count the unique number of people who filled in the first survey page (or agreed to participate, for example by checking a checkbox), divided by visitors who visit the first page of the survey (or the informed consents page, if present).*

| **Survey** | **Participation rate** |
| --- | --- |
| HCP international | 157/2860 = 5% |
| HCP Netherlands | 551/2250 = 24% |
| Pregnant women International | 205/1082 = 19% |
| Pregnant women Netherlands | 714/1566 = 46% |

Completion rate

*The number of people submitting the last questionnaire page, divided by the number of people who agreed to participate (or submitted the first survey page.*

| **Survey** | **Completion rate** |
| --- | --- |
| HCP international | 92/157 = 59% |
| HCP Netherlands | 404/551 = 73% |
| Pregnant women International | 141/205 = 69% |
| Pregnant women Netherlands | 488/714 = 68% |

Survey progress rate

| **Rate of survey completion (%)** | **Healthcare practitioners** | **Pregnant women** |
| --- | --- | --- |
| 0-40 | 45 (75) | 0 (0%) |
| 41-60 | 41 (7%) | 54 (7%) |
| 61-80 | 20 (3%) | 37 (5%) |
| 81-100 | 502 (83%) | 703 (88%) |

**6 – Sub-analysis per subspecialism**

| **Statement** | **Community pharmacists** | **Clinical pharmac(olog)ists** | **Obstetricians-gynaecologists** | **General practitioners** | **Other medical specialists*** |
| --- | --- | --- | --- | --- | --- |
| I regularly adjust or recommend adjustments in doses of pregnant patients | N = 151: 62% agreed | N = 67: 55% agreed | N = 151: 48% agreed | N = 57: 52% agreed | N = 96: 74% agreed |
| I have sufficient understanding of how pregnancy can influence the pharmacokinetics of medications and of how this may result in an altered safety and effectiveness profile during pregnancy | N = 144: 40% agreed | N = 68: 51% agreed | N = 149: 34% agreed | N = 57: 39% agreed | N = 76: 33% agreed |
| I would be willing to follow dose recommendations for pregnant women that are primarily based on evidence from computer models | N = 157: 90% agreed | N = 75: 92% agreed | N = 155: 97% agreed | N = 59: 88% agreed | N = 93: 88% agreed |
| Fetal safety is a bigger concern than maternal effectiveness when I prescribe medication or give advice on medication to a pregnant woman that requires medication | N = 153: 82% agreed | N = 68: 62% agreed | N = 152: 49% agreed | N = 58: 74% agreed | N = 89: 51% agreed |
| I am willing to advise or prescribe a higher medication dose to a pregnant woman if this is recommended by the pregnancy formulary, including if the main evidence stems from pharmacokinetic models | N = 134: 79% agreed | N = 61: 89% agreed | N = 143: 90% agreed | N = 53: 91% agreed | N = 79: 92% agreed |

*Other medical specialists include paediatricians/neonatologists, internists, psychiatrists, cardiologists, neurologists, anaesthesiologists, dermatologists and gastroenterologists.

**7 – Statement ratings (non-trichotomized)**

**7.1 HCPs**

| **Statement** | **Strongly disagree** | **Somewhat disagree** | | **Somewhat agree** | **Strongly agree** | **Not applicable** |
| --- | --- | --- | --- | --- | --- | --- |
| I would be willing to follow dose recommendations for pregnant women that are primarily based on evidence from computer models (N = 608) | 17 (3%) | 23 (4%) | | 367 (60%) | 193 (32%) | 7 (1%) |
| Fetal safety is a bigger concern than the maternal effectiveness when I prescribe medication or give advice on medication to a pregnant woman that requires medication (N = 588) | 27 (5%) | 163 (28%) | | 296 (50%) | 98 (16%) | 4 (1%) |
| I regularly use Lareb’s website to obtain information on the fetal safety of medication (NL only, N = 464) | 51 (11%) | 25 (5%) | | 71 (15%) | 317 (68%) | 4 (1%) |
| I regularly adjust or recommend adjustments in the medication doses of pregnant patients or patients that wish to be pregnant (N = 577) | 94 (16%) | 136 (24%) | | 215 (37%) | 109 (19%) | 23 (4%) |
| I generally discuss my considerations for choosing a medication dose with my pregnant patients (N = 569) | 58 (10%) | 68 (12%) | | 186 (33%) | 230 (40%) | 27 (5%) |
| I have a good enough understanding of how pregnancy can influence the pharmacokinetics (absorption, distribution, metabolism, elimination) of medications and how this may result in an altered safety and effectiveness profile during pregnancy (N = 566) | 126 (22%) | 220 (39%) | | 176 (31%) | 42 (7%) | 2 (1%) |
| I know what pharmacokinetic models entail (N = 567) | 57 (10%) | 134 (24%) | | 257 (45%) | 119 (21%) | NA |
| There is a need for better information on the fetal safety of medications (N = 560) | 5 (1%) | 27 (5%) | | 155 (28%) | 373 (66%) | NA |
| There is a need for better information on medication dosing in pregnancy (N = 557) | 3 (1%) | 15 (3%) | | 104 (18%) | 435 (78%) | NA |
| I am concerned that pregnant women receive suboptimal pharmacological care (N = 557) | 18 (3%) | 76 (14%) | | 274 (49%) | 189 (34%) | NA |
| The availability of an evidence-based pregnancy formulary would greatly enhance the quality of care for pregnant women (N = 557) | 8 (1%) | 9 (2%) | | 129 (23%) | 411 (74%) | NA |
| I am willing to advise or prescribe a higher medication dose to a pregnant woman if this is recommended by the pregnancy formulary, including if the main evidence stems from pharmacokinetic models (N = 532) | Disagree: 59 (11%) | | | Agree: 451 (85%) | | 22 (4%) |
| I am willing to advise or prescribe a higher medication dose to a pregnant woman if this is recommended by the pregnancy formulary, including if the fetal exposure to the medication is unknown (N = 526) | 75 (14%) | 172 (33%) | | 211 (40%) | 49 (9%) | 19 (4%) |
| I would be more willing to follow dose recommendations in the pregnancy formulary if the formulary is endorsed by recognized institutions and professional associations (N = 525) | 8 (2%) | 7 (1%) | | 130 (25%) | 373 (21%) | 7 (1%) |
| Do you agree with the following? I have a preference for framing A over framing B (N = 509)  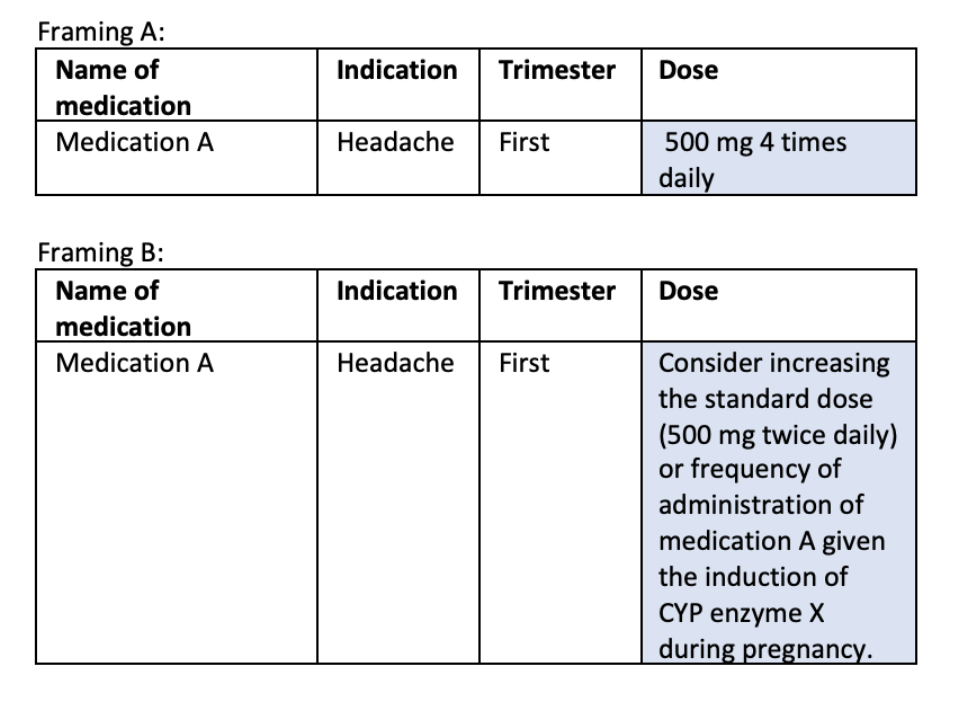 | Strong preference for framing A: 65 (13%) | Slight preference for framing A: 80 (16%) | | Slight preference for framing B: 162 (32%) | Strong preference for framing B: 199 (39%) | NA |
| I would like the following information to be included in the pregnancy formulary alongside the dose recommendation for a given medication: | | | | | | |
| How the risks associated with the dose recommendation compare with the benefits of said dose recommendation for pregnant women and their unborn child (N = 500) | 7 (1%) | 9 (2%) | | 156 (31%) | 328 (66%) | NA |
| Relevant physiological changes in pregnancy and how they affect the pharmacokinetics of that medication (N = 504) | 9 (2%) | 37 (7%) | | 189 (38%) | 269 (55%) | NA |
| Information on the fetal exposure of the medication  (N = 504) | 9 (2%) | 32 (6%) | | 161 (32%) | 302 (60%) | NA |
| Information on the fetal safety of the medication (N = 504) | 4 (1%) | 4 (1%) | | 56 (11%) | 440 (87%) | NA |
| Recommendations on how to adjust the recommended dose based on the individual characteristics of a pregnant patient (e.g., high body mass index) (N = 504) | 6 (1%) | 6 (1%) | | 118 (25%) | 369 (73%) | NA |
| Recommendations for detecting the occurrence of underdosing or toxicity in a pregnant patient receiving the recommended dose (e.g., through therapeutic drug monitoring) (N = 503) | 10 (2%) | 22 (4%) | | 179 (36%) | 292 (58%) | NA |
| I am likely to consult information on the evidence behind a dose recommendation in pregnancy (N = 502) | Disagree: 58 (12%) | | | Agree: 444 (88%) | | NA |
| What I would like to know: | | | | | | |
| What the quality of the underlying evidence is (N = 441) | 4 (1%) | 6 (1%) | | 129 (29%) | 302 (69%) | NA |
| I would like to have access to the evidence itself (N = 442) | 14 (3%) | 80 (18%) | | 205 (47%) | 143 (32%) | NA |
| If the evidence for a dose recommendation comes from a pharmacokinetic model, I would like to have access to information on the model (N = 441) | Disagree: 168 (33%) | | | Agree: 273 (62%) | | NA |
| The general assumptions of the model (N = 273) | 3 (1%) | 8 (3%) | | 133 (49%) | 129 (47%) | NA |
| Information on how fetal exposure was determined (N = 273) | 5 (2%) | 13 (5%) | | 112 (41%) | 143 (51%) | NA |
| Information on model validation (N = 273) | 5 (2%) | 25 (9%) | | 122 (45%) | 121 (44%) | NA |
| I would like to have access to the model itself (N = 273) | 48 (18%) | 83 (30%) | | 101 (37%) | 41 (15%) | NA |
| My preference would be to access the pregnancy formulary on a new stand-alone website (e.g. pregnancyformulary.com) (N = 499) | 60 (12%) | 109 (22%) | | 166 (33%) | 164 (32%) | NA |
| My preference would be for the pregnancy formulary to be housed on a website that I already use (N = 499) | Disagree: 188 (32%) | | | Agree: 311 (62%) | | NA |
| I would like the pregnancy formulary to be added to the Lareb website (NL only, N = 268) | 15 (6%) | 20 (7%) | | 71 (26%) | 162 (61%) | NA |
| I would like the pregnancy formulary to be added to the Dutch National Formulary (NL only, N = 268) | 23 (9%) | 32 (12%) | | 77 (29%) | 136 (50%) | NA |
| I would like to have access to the pregnancy formulary via a mobile app (N = 499) | 25 (5%) | 64 (13%) | | 158 (32%) | 252 (50%) | NA |
| I would like to be able to refer a pregnant woman to online patient information about dosing in pregnancy (N = 499) | 19 (4%) | 46 (9%) | | 159 (32%) | 275 (55%) | NA |
| Key considerations for including medications in the pregnancy formulary should be (maximum 2 responses, 733 responses in total): | The frequency of use of the medication among pregnant women: 373 (50%) | The number of specialties prescribing the medication to pregnant women: 41 (6%) | | | The consequences of underdosing or overdosing in pregnancy: 322 (44%) | |
| I would like to be part of the editorial board of the envisioned pregnancy formulary (N = 406) | No: 343 (84%) | | Yes: 63 (16%) | | | |

**7.2. Pregnant women**

| **Statement** | **Strongly disagree** | **Somewhat disagree** | **Somewhat agree** | **Strongly agree** |  |
| --- | --- | --- | --- | --- | --- |
| I would like to know whether I can safely use a medication during pregnancy (N = 794) | Disagree: 6 (1%) | | Agree: 788 (99%) | |  |
| I would like to know whether I can safely use a medication during breastfeeding (N = 789) | Disagree: 21 (3%) | | Agree: 768 (97%) | |  |
| I want to know about the evidence that was used to determine if a medication can safely be used during my pregnancy or breastfeeding (N = 781) | 20 (3%) | 37 (5%) | 223 (29%) | 501 (63%) |  |
| I have looked up information on whether I can safely use a medication during pregnancy or breastfeeding (N = 785) | Disagree: 45 (6%) | | Agree: 572 (94%) | |  |
| I could easily find clear and helpful information about the safety of medication during pregnancy or breastfeeding (N = 728) | 98 (13%) | 244 (32%) | 321 (44%) | 65 (9%) |  |
| I have already consulted the Lareb Mothers of Tomorrow website to look up information about medications during pregnancy or breastfeeding (N = 564) | Disagree: 379 (67%) | | Agree: 185 (33%) | |  |
| The effectiveness of the medication I use while I am pregnant, or breastfeeding is as important to me than the safety of this medication for my baby (N = 767) | Disagree, the efficacy of my medication is more important: 1 (1%) | Disagree, the safety of my baby is more important: 388 (50%) | Agree: 379 (49%) | | |
| I want to be involved in decisions on which medications I should use during pregnancy or breastfeeding together by my healthcare practitioner (N = 763) | 10 (1%) | 18 (2%) | 180 (10%) | 555 (73%) |  |
| Before I read the introduction of this survey, I was aware that changes in my body during pregnancy can influence the dose of medication I need (N = 744) | 155 (21%) | 122 (16%) | 179 (24%) | 288 (39%) |  |
| I am aware that the doses of medication used in pregnant women are often based on research from studies conducted in people who are not pregnant (N = 743) | 230 (31%) | 130 (17%) | 191 (26%) | 192 (26%) |  |
| I would like to know which medication dose I should use during pregnancy (N = 730) | 5 (1%) | 5 (1%) | 53 (7%) | 667 (92%) |  |
| I would like to know which medication dose I should use during breastfeeding (N = 726) | 21 (3%) | 5 (1%) | 61 (8%) | 639 (88%) |  |
| I have looked up information about which dose of medication I should use during pregnancy (N = 725) | Disagree: 257 (35%) | | Agree: 468 (65%) | |  |
| I could easily find clear and helpful information about a medication dose during pregnancy (N = 463) | 122 (26%) | 158 (34%) | 150 (32%) | 33 (7%) |  |
| I would like to understand why pregnant women may sometimes need a higher or a lower dose of medication than women that are not pregnant (N = 716) | 7 (1%) | 23 (3%) | 199 (28%) | 487 (68%) |  |
| I would like to have information on how much of the medication I use during my pregnancy goes to my baby (N = 715) | 7 (1%) | 19 (3%) | 135 (19%) | 554 (77%) |  |
| I want to be involved in decisions on which dose of a medication I should use during pregnancy by my healthcare practitioner (N = 713) | 6 (1%) | 17 (2%) | 158 (22%) | 532 (77%) |  |
| I am willing to use a higher dose of medication during my pregnancy than that I would receive if I was not pregnant, if my healthcare practitioner recommends this (N = 706) | Disagree: 128 (18%) | | Agree: 578 (82%) | |  |
| If I was advised to use a higher dose, I would be concerned about the safety of this dose for my baby (N = 127) | 0 (0%) | 2 (2%) | 18 (14%) | 107 (84%) |  |
| I would be willing to use an increased dose of medication if my healthcare practitioner explains the reasons behind the chosen dose (N = 127) | 2 (2%) | 16 (13%) | 69 (54%) | 40 (31%) |  |
| I am willing to have a small blood sample taken before and during my pregnancy if this helps to make sure that I receive the right medication dose for treating my condition (N = 703) | 4 (1%) | 16 (2%) | 68 (10%) | 615 (87%) |  |
| I am willing to use a medication dose that was determined by computer models alongside the expertise of medical professionals (N = 663) | Disagree: 156 (24%) | | Agree: 507 (76%) | |  |
| One reason that I am in favour of using computer models is that they help determine the right medication dose for treating me effectively during pregnancy (N = 492) | 2 (1%) | 20 (4%) | 289 (59%) | 181 (36%) |  |
| Another reason that I am in favour of using such models is that they give information on how much of the medication I receive goes to my unborn child (N = 484) | 2 (1%) | 16 (3%) | 219 (45%) | 247 (51%) |  |
| I would like to know more about how these computer models work (N = 636) | 29 (5%) | 60 (9%) | 188 (30%) | 359 (56%) |  |
| I would like to have more information about how changes in my body during pregnancy affect the amount of medication I need (N = 634) | 5 (1%) | 15 (2%) | 172 (27%) | 442 (70%) |  |
| I would like my healthcare practitioner to share this information with me (N = 632) | 3 (1%) | 15 (2%) | 139 (22%) | 475 (75%) |  |
| I would like to get this information through a website (N = 631) | 9 (1%) | 631 (4%) | 180 (29%) | 419 (66%) |  |
